# Ovalbumin with Glycated Carboxyl Groups Shows Membrane-Damaging Activity

**DOI:** 10.3390/ijms18030520

**Published:** 2017-02-28

**Authors:** Ching-Chia Tang, Yi-Jun Shi, Ying-Jung Chen, Long-Sen Chang

**Affiliations:** 1 Institute of Biomedical Sciences, National Sun Yat-Sen University, Kaohsiung 804, Taiwan; jenny.tang0723@gmail.com (C.-C.T.); a786514@gmail.com (Y.-J.S.); 2 Department of Fragrance and Cosmetic Science, Kaohsiung Medical University, Kaohsiung 807, Taiwan; yjchen@kmu.edu.tw; 3 Department of Biotechnology, Kaohsiung Medical University, Kaohsiung 807, Taiwan

**Keywords:** ovalbumin, glycation, carboxyl group, membrane-damaging activity

## Abstract

The aim of the present study was to investigate whether glycated ovalbumin (OVA) showed novel activity at the lipid-water interface. Mannosylated OVA (Man-OVA) was prepared by modification of the carboxyl groups with *p*-aminophenyl α-dextro (d)-mannopyranoside. An increase in the number of modified carboxyl groups increased the membrane-damaging activity of Man-OVA on cell membrane-mimicking vesicles, whereas OVA did not induce membrane permeability in the tested phospholipid vesicles. The glycation of carboxyl groups caused a notable change in the gross conformation of OVA. Moreover, owing to their spatial positions, the Trp residues in Man-OVA were more exposed, unlike those in OVA. Fluorescence quenching studies suggested that the Trp residues in Man-OVA were located on the interface binds with the lipid vesicles, and their microenvironment was abundant in positively charged residues. Although OVA and Man-OVA showed a similar binding affinity for lipid vesicles, the lipid-interacting feature of Man-OVA was distinct from that of OVA. Chemical modification studies revealed that Lys and Arg residues, but not Trp residues, played a crucial role in the membrane-damaging activity of Man-OVA. Taken together, our data suggest that glycation of carboxyl groups causes changes in the structural properties and membrane-interacting features of OVA, generating OVA with membrane-perturbing activities at the lipid-water interface.

## 1. Introduction

The structure-function relationship of proteins has been extensively studied using chemical modification or site-directed mutagenesis. The loss in biological activity allows the researchers to determine the crucial residues involved in the biological functions of proteins. However, some studies have shown that covalent modification of proteins could modulate the activity and structure of proteins [1]. Chemical modification of carboxyl groups increases the enzymatic activity of *Candida* *antarctica* and *Thermomyces lanuginose* lipases [2]. Modification of lysine residues in *Bacillus licheniformis* α-amylase changes its substrate specificity [3]. These results suggest that chemical modification could improve the biological activities of proteins. Nevertheless, limited studies have made an effort to address the possibility that chemical modification confers novel activities to proteins, owing to the lack of a rational approach for identifying the unknown activities of chemically-modified proteins. Previous studies have revealed that bovine serum albumin (BSA) could transfer lipid amphiphile to lipid bilayer membranes [4], suggesting that BSA could interact with lipid bilayers. Accordingly, one may imagine that chemical modification potentially causes a change in the membrane-interacting mode of BSA. Although BSA does not demonstrate membrane-perturbing activity, mannosylated bovine serum albumin (Man-BSA) prepared by the conjugation of carboxyl groups with *p*-aminophenyl α-dextro (d)-mannopyranoside, exhibits membrane-damaging activity on the lipid-water interface [5]. These results suggest that protein with a membrane-binding capability can show membrane-perturbing activities after the glycation of its carboxyl groups. To test whether the methodology is common for the preparation of proteins with novel activities on the lipid-water interface, further studies on other proteins with membrane-binding properties might throw more light on this suggestion.

Chicken egg-white is widely used as an ingredient in the food industry, to enhance and improve the functionality of various food products [6]. Ovalbumin (OVA) is a glycoprotein that is present in large amounts in the avian egg white. It is a 385 amino acid polypeptide chain with a single N-linked glycosylation site at Asn292 [7]. The protein is cross-linked by one disulfide bond (Cys73–Cys120) and has four Cys residues at positions 11, 30, 367, and 382, with free sulfhydryl groups [8]. OVA is grouped into the serpin (serine protease inhibitor) superfamily because of its primary and tertiary structure, but it does not inhibit serine proteases [9]. Glycated OVA prepared by a Maillard reaction exhibits anti-oxidant activity [10]. The Maillard reaction involves condensation of the sugar with the amino groups of proteins [11], suggesting that the modification of amino groups could alter the functional properties of OVA. In addition, Li et al. [12] reported that 3-hydroxyphthalic anhydride-, maleic anhydride-, and succinic anhydride-modified OVA exhibit anti-HIV activity, which positively correlates with the percentage of chemically-modified Lys and Arg residues in the modified OVA. These observations indicate that chemical modification confers novel properties to OVA. Notably, whether modification of carboxyl groups can induce OVA to display new biological activities, has not yet been studied. Yun and Kim [13] reported that OVA induces the fusion and leakage of phosphatidylserine (PS)/phosphatidylethanolamine (PE) vesicles in acidic solutions, but the membrane-perturbing activities of OVA are not observed when the pH is >6. Considering that the pI value of OVA is approximately 4.7, it may be positively charged in an acidic solution, and thus favor interaction with negatively charged phospholipids. Similarly, the removal of negatively charged carboxyl groups may enable OVA to display membrane-perturbing activities in a solution at a physiological pH. To test this proposition, the membrane-perturbing activities of mannosylated OVA (Man-OVA), prepared by conjugating carboxyl groups with *p*-aminophenyl α-d-mannopyranoside, were investigated in the present study. The results of the present study confirm that the glycation of carboxyl groups changes the membrane-interacting feature of OVA.

## 2. Results

Previous studies have shown that Man-BSA, prepared by the conjugation of carboxyl groups with *p*-aminophenyl α-d-mannopyranoside, demonstrates membrane-damaging activity [5]. Therefore, *p*-aminophenyl α-d-mannopyranoside was used to modify carboxyl groups in OVA. OVA was conjugated with increasing concentrations of *p*-aminophenyl α-d-mannopyranoside at molar ratios of 1:50, 1:150, 1:300, and 1:600, and the resulting mannosylated OVAs (Man-OVA) were designated as Man-OVA(50), Man-OVA(150), Man-OVA(300), and Man-OVA(600), respectively. As shown in Figure S1A, the electrophoretic mobility of Man-OVA(50), Man-OVA(150), Man-OVA(300), and Man-OVA(600) on SDS-PAGE, was slower than that of OVA. In the matrix-assisted laser desorption ionization-time of flight (MALDI-TOF) analysis, compared with that of OVA, the molecular weight of Man-OVA(50), Man-OVA(150), Man-OVA(300), and Man-OVA(600) was higher, by 3283, 4786, 5986, and 7758 Da, respectively (Figure S1B, supplementary materials). Thus, the conjugation of approximately 13, 19, 24, and 31 *p*-aminophenyl α-d-mannopyranoside with the carboxyl groups, was calculated from the increment in the molecular weight of Man-OVA(50), Man-OVA(150), Man-OVA(300), and Man-OVA(600), respectively. Because OVA contains a 14 Asp, 32 Glu, and 1 C-terminal carboxyl group, it can be inferred that the conjugation did not modify all of the carboxyl groups in OVA. In addition, the number of modified carboxyl groups did not show a linear relationship with increasing *p*-aminophenyl α-d-mannopyranoside concentrations, suggesting that each of the carboxyl groups of OVA reacted differently with *p*-aminophenyl α-d-mannopyranoside. As illustrated in Figure S1C, Man-OVA showed absorbance at 260 nm, owing to the incorporated aminophenyl groups. Reverse phase high performance liquid chromatography (HPLC) analysis revealed that Man-OVA eluted earlier than OVA (Figure S1D). These results demonstrate that *p*-aminophenyl α-d-mannopyranoside was successfully conjugated with OVA. The circular dichroism (CD) spectra of Man-OVA and OVA differed (Figure S1E), indicating that glycation caused a change in the gross structure of OVA.

**Figure 1:**
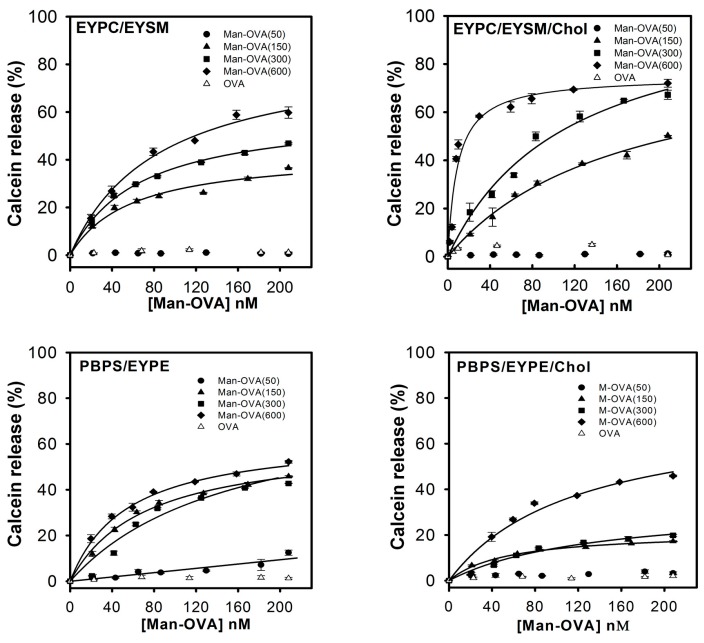
Membrane-perturbing activity of ovalbumin (OVA) and Man-OVA on EYPC/EYSM, EYPC/EYSM/Chol, PBPS/EYPE, and PBPS/EYPE/Chol vesicles. The experiments were performed in 10 mM Tris–HCl-0.1 M NaCl (pH 7.5). The concentrations of phospholipid vesicles were 15 μM.

Previous studies have revealed that OVA induces leakage from PS/PE vesicles in acidic solutions [8]. PS and PE are generally enriched in the inner leaflet, while phosphatidylcholine (PC), sphingomyelin (SM), and glycosphingolipids, are predominant in the outer leaflet of the cell membrane [14]. Moreover, cholesterol (Chol) is universally present in large amounts (20–40 mol %), in both the outer and inner leaflets of the cell membrane [15]. Therefore, the phospholipid vesicles composed of egg yolk phosphatidylcholine (EYPC)/egg yolk sphingomyelin (EYSM) (53/47, mol/mol), EYPC/EYSM/Chol (37/33/30, mol/mol/mol), porcine brain phosphatidylserine (PBPS)/egg yolk phosphatidylethanolamine (EYPE) (50/50, mol/mol), and PBPS/EYPE/Chol (35/35/30, mol/mol/mol), which mimicked the properties of the outer or inner leaflets of the cell membrane [16,17], were used to study the membrane-damaging activities of Man-OVA and OVA. Figure 1 shows that Man-OVA(150), Man-OVA(300), and Man-OVA(600) induced a dose-dependent leakage in all four types of vesicles. However, Man-OVA(50) was unable to increase the permeability of EYPC/EYSM, EYPC/EYSM/Chol, and PBPS/EYPE/Chol vesicles, but marginally induced that of PBPS/EYPE vesicles. A maximal calcein release from all four types of vesicles was observed when using Man-OVA(150), Man-OVA(300), and Man-OVA(600), at concentrations >200 nM. Moreover, the membrane-damaging activity of Man-OVA(600) on all tested vesicles was higher than that noted for Man-OVA(150) and Man-OVA(300). These results suggested that an increase in the number of modified carboxyl groups promoted the membrane-perturbing activity of Man-OVA. In contrast, OVA did not induce membrane permeability in the tested vesicles. Because Man-OVA(600) showed the highest membrane-damaging activity, this Man-OVA was employed to further explore the mechanism of glycation-induced membrane damage. Man-OVA(600) induced the maximal calcein release of approximately 59.7%, 71.5%, 52.2%, and 45.9%, from EYPC/EYSM, EYPC/EYSM/Chol, PBPS/EYPE, and PBPS/EYPE/Chol vesicles, respectively. This result indicated that the lipid composition might affect the membrane-damaging activity of Man-OVA.

**Figure 2:**
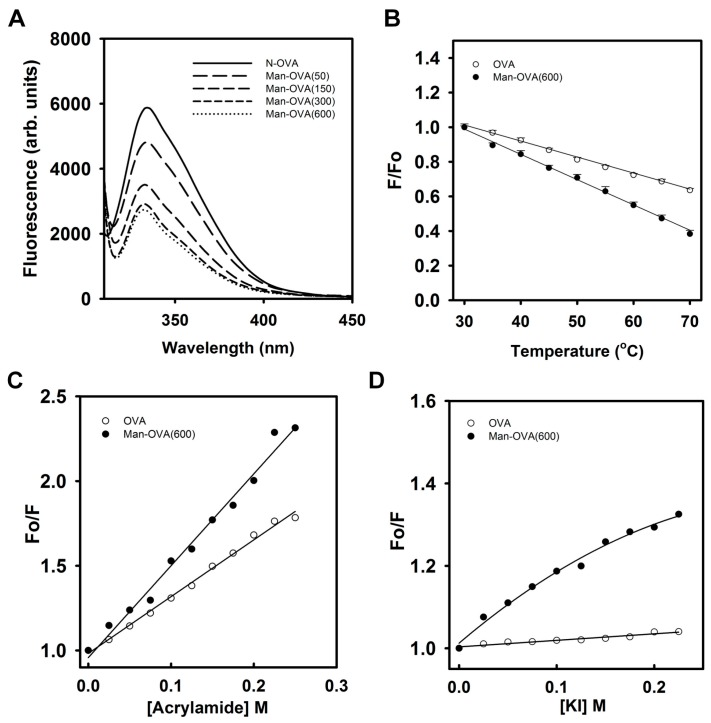
Man-OVA and OVA have different Trp fluorescence spectra, thermal stability, and susceptibility for acrylamide and KI quenching. The experiments were measured using an exciting wavelength at 295 nm, and the protein concentration was 0.1 μM. (**A**) Intrinsic fluorescence spectra of OVA and Man-OVA; (**B**) Effect of temperature on Trp fluorescence of OVA and Man-OVA(600). Trp fluorescence quenching of OVA and Man-OVA(600) by acrylamide (**C**) and iodide (**D**).

Figure 2A shows that the conjugation of carboxyl groups with *p*-aminophenyl α-d-mannopyranoside reduced the Trp fluorescence intensity of OVA, suggesting that glycation of OVA altered the microenvironment of its Trp residues [18]. Meanwhile, titration of OVA with *p*-aminophenyl α-d-mannopyranoside marginally reduced its Trp fluorescence intensity (Figure S2). This result indicated that the incorporated glycated groups might not quench the Trp fluorescence of OVA. As shown in Figure 2B, the reduction in the Trp fluorescence of Man-OVA(600) with an increase in temperature was greater than that of OVA, suggesting that the structural stability of Man-OVA(600) was lower than that of OVA. Linear Stern–Volmer plots obtained for OVA and Man-OVA(600) revealed that all of the Trp residues in respective proteins are equally accessible to acrylamide (Figure 2C). However, the susceptibility of the Trp residues in Man-OVA(600) to acrylamide was higher than that of the residues in OVA, suggesting that the spatial positional changes caused by glycation caused the Trp residues in OVA to become more exposed. Previous studies have shown that iodide quenching could reveal whether the Trp residues in proteins were located in a positively charged environment [19]. Therefore, iodide quenching of Man-OVA and OVA was analyzed using the Stern-Volmer plot. A linear plot for OVA in Figure 2D indicates an equal accessibility of all Trp residues to iodide. However, the downward curve observed for Man-OVA(600) is suggestive of two possible classes of Trp fluorophores [19], of which one is more accessible to iodide than the other. Nevertheless, the accessibility of the Trp residues in Man-OVA(600) to iodide was higher than that of the residues in OVA, suggesting that the microenvironment of the Trp residues in Man-OVA(600) contained a greater number of positively charged residues than that of OVA. This is consistent with the fact that the conjugation of carboxyl groups with *p*-aminophenyl α-d-mannopyranoside decreases the number of negatively charged residues in OVA.

Because the microenvironment of the Trp residues in OVA was affected by the glycation of carboxyl groups, we explored the role of Trp residues in the interaction of Man-OVA(600) with phospholipid vesicles. As listed in Table 1, the accessibility of Man-OVA to acrylamide was reduced in the presence of EYPC/EYSM, EYPC/EYSM/Chol, PBPS/EYPE, and PBPS/EYPE/Chol vesicles, suggesting that the Trp residues of Man-OVA(600) were located at the interface that binds phospholipid vesicles. Similarly, the accessibility of the Trp residues in OVA to acrylamide was also reduced in the presence of phospholipid vesicles (Table 1), indicating that OVA was able to bind phospholipid vesicles. This is consistent with previous studies showing that OVA can absorb onto the surface of the 1,2-dipalmitoyl-sn-glycero-3-phosphocholine monolayer [20].

**Figure 3:**
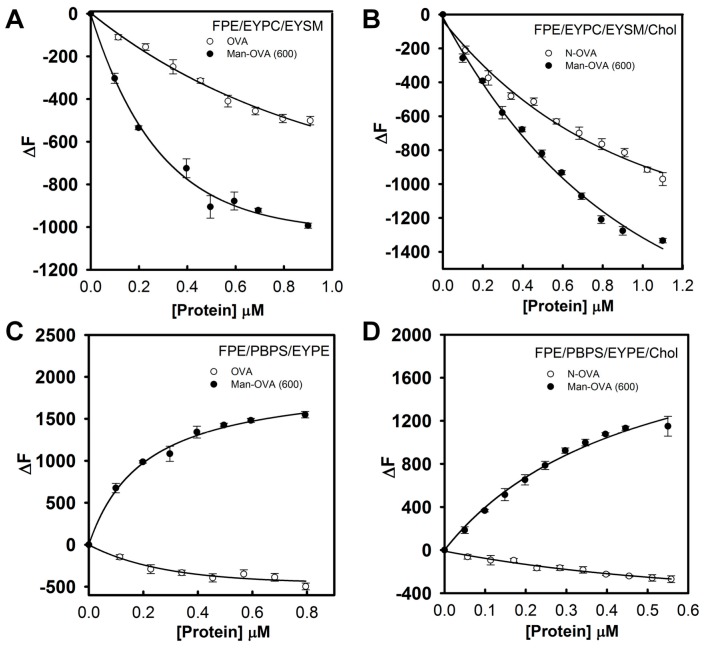
The binding capability of OVA and Man-OVA(600) with phospholipid vesicles. Binding of OVA and Man-OVA(600) with phospholipid vesicles enhanced the fluorescence intensity of (**A**) FPE/EYPC/EYSM; (**B**) FPE/EYPC/EYSM/Chol; (**C**) FPE/PBPS/EYPE; and (**D**) FPE/PBPS/EYPE/Chol vesicles. The lipid concentration was 81.4 μM. The fluorescence emission intensity at 510 nm was measured.

The fluorescence intensity of *N*-(fluorescein-5-thiocarbamoyl)-1,2-dihexadecanoyl-phosphatidylethanolamine (FPE) is sensitive to the ionization state of the carboxyl group of fluorescein, and it increases when the negative surface charge of the membrane is reduced by the binding of a protein [21]. In contrast, the addition of proteins with a negative charge increases the membrane’s negative electrostatic potential, causing a reduction in FPE fluorescence [22]. As shown in Figure 3A,B, the fluorescence intensity of FPE in FPE/EYPC/EYSM and FPE/EYPC/EYSM/Chol vesicles decreased with increasing Man-OVA or OVA concentrations. However, Man-OVA(600) caused a greater reduction in FPE fluorescence than OVA. The binding of OVA to FPE/PBPS/EYPE and FPE/PBPS/EYPE/Chol vesicles reduced the FPE fluorescence intensity, whereas the binding of Man-OVA(600) enhanced its intensity (Figure 3C,D). These findings suggested that Man-OVA(600) and OVA adopted different topographical arrangements upon absorption on EYPC/EYSM, EYPC/EYSM/Chol, PBPS/EYPE, and PBPS/EYPE/Chol vesicles. The binding affinities of Man-OVA and OVA for lipid vesicles were determined by the changes in FPE fluorescence. The dissociation constants (K_d_) of Man-OVA for EYPC/EYSM, EYPC/EYSM/Chol, PBPS/EYPE, and PBPS/EYPE/Chol vesicles, were 0.38 ± 0.05, 0.60 ± 0.02, 0.26 ± 0.04, and 0.67 ± 0.03 μM, respectively. The K_d_ values of OVA for EYPC/EYSM, EYPC/EYSM/Chol, PBPS/EYPE, and PBPS/EYPE/Chol vesicles, were 0.90 ± 0.03, 0.30 ± 0.04, 0.43 ± 0.03, and 0.27 ± 0.02 μM, respectively. Evidently, the inability of OVA to damage the membrane was not related to its ability to bind phospholipid vesicles.

**Figure 4:**
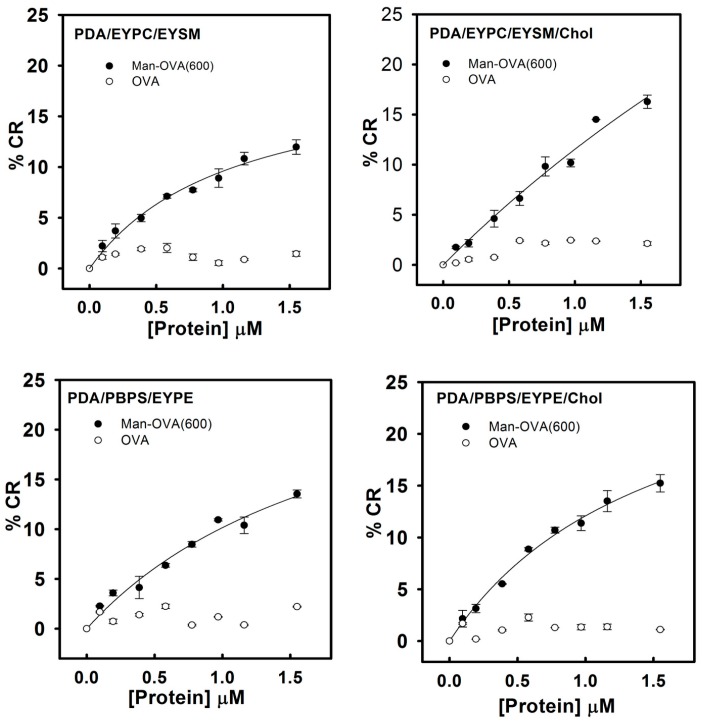
Colorimetric dose-response curve of PDA/EYPC/EYSM, PDA/EYPC/EYSM/Chol, PDA/PBPS/EYPE, and PDA/PBPS/EYPE/Chol vesicles, titrated with Man-OVA(600) and OVA. The experiments were conducted according to the procedure described in the Materials and Methods section. The total lipid concentration of the PDA/EYPC/EYSM, PDA/EYPC/EYSM/Chol, PDA/PBPS/EYPE, and PDA/PBPS/EYPE/Chol solution was 0.5 mM.

Previous studies have indicated that lipid/polydiacetylene (PDA) vesicles mimic the environment in the cell membranes and provide information on the binding of pore-forming peptides with membranes [23,24]. The blue-to-red transformation in lipid/PDA vesicles depends on the disruption of the interface and structure of the lipid bilayer by membrane-interacting peptides [18]. Peptides that preferably disrupted the lipid head-group region or penetrated the hydrophobic lipid core induced pronounced color transitions [24,25]. Therefore, a higher colorimetric response (%CR) reflected the higher ability of the membrane-interacting peptides to perturb the membrane structure. As shown in Figure 4, compared to OVA, Man-OVA(600) induced a notable increase in the %CR of PDA/EYPC/EYSM, PDA/EYPC/EYSM/Chol, PDA/PBPS/EYPE, and PDA/PBPS/EYPE/Chol vesicles, suggesting that distinct structural features of OVA and Man-OVA(600) were in contact with the interface of EYPC/EYSM, EYPC/EYSM/Chol, PBPS/EYPE, and PBPS/EYPE/Chol vesicles.

**Figure 5:**
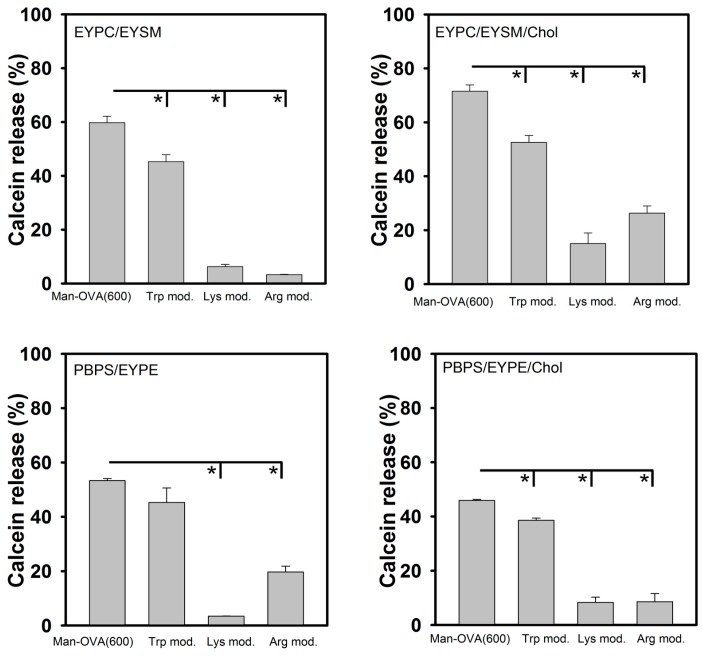
Membrane-damaging activity of Trp-, Lys-, and Arg-modified Man-OVA(600). Man-OVA(600) and modified Man-OVA(600) induced calcein release from EYPC/EYSM, EYPC/EYSM/Chol, PBPS/EYPE, and PBPS/EYPE/Chol vesicles (* *p* < 0.05). The concentrations of protein and lipid vesicles were 0.2 and 15 μM, respectively.

Our findings show that the microenvironment of the Trp residues of Man-OVA(600) was enriched in positively charged residues and that these residues are located at the interface of Man-OVA and the interacting lipid vesicles. Therefore, the role of Trp, Lys, and Arg residues in the membrane-damaging activity of Man-OVA(600), was further analyzed using chemical modification. The results of amino acid analysis showed that the three Trp residues in Man-OVA(600) (positions 149, 185, and 286 in OVA) were modified with *N*-bromosuccinimide, and that trinitrobenzene sulfonate (TNBS) and phenylglyoxal specifically modified Lys and Arg residues, respectively. MALDI-TOF analyses of Lys- and Arg-modified Man-OVA(600) showed an incorporation of six trinitrophenyl and four phenylglyoxal groups, respectively (data not shown). As shown in Figure 5, compared with that of Man-OVA(600), a marked reduction in membrane-damaging activity was observed with Lys- and Arg-modified Man-OVA(600). In contrast, Trp modification reduced the membrane-damaging activity of Man-OVA(600) to a lesser extent, indicating that the positively charged residues of Man-OVA(600) were crucial for its membrane-damaging activity.

## 3. Discussion

This study shows that the modification of carboxyl groups with *p*-aminophenyl α-d-mannopyranoside generates glycated OVA with membrane-damaging activity. Man-OVA and OVA bind phospholipid vesicles with similar affinity. Unlike Man-OVA, OVA does not exhibit membrane-damaging activity in Tris buffer (pH 7.5). This is consistent with previous reports that OVA could not induce membrane permeability in solutions with pH > 6 [13]. The interaction with PDA-containing vesicles revealed that the structural feature of Man-OVA that interacts with lipid vesicles is distinct from that of OVA. CD spectra and fluorescence measurements showed that the glycation of OVA causes a change in its gross conformation. Chemical modification studies revealed that an increase in positive characters due to the blocking of negatively charged carboxyl groups contributes to the membrane-damaging activity of Man-OVA. In conclusion, the glycation of carboxyl groups in OVA simultaneously changes its structural properties and membrane-interaction mechanism, thereby conferring the membrane-perturbing activity.

Previous studies reported that OVA only induces leakage in PS/PE vesicles in acidic solutions [13]. Koseki et al. [26] observed that the structure of OVA is more flexible and susceptible to denaturation at an acidic pH, and suggested that this structural flexibility and instability may enable OVA to damage membranes in acidic solutions. El Amri et al. [27] proposed that the structural flexibility of plasticins, membrane-damaging peptides from frog skin, modulates their ability to disrupt the membrane bilayers. Kim et al. [28] reported that the structural flexibility of piscidin 1, a cytotoxic peptide isolated from the mast cells of hybrid striped bass, is important for its ability to penetrate membranes. Because the structural stability of Man-OVA(600) was lower than that of OVA, it is likely that the less-ordered structure of Man-OVA plays a role in its membrane-damaging activity. In contrast, the modification of Lys or Arg residues causes a marked decrease in the membrane-damaging activity of Man-OVA. Although an increase in positively charged residues might favor an interaction with anionic phospholipids, Man-OVA induces the membrane permeability of vesicles composed of anionic phospholipids (EYPS), as well as zwitterionic phospholipids (EYPC, EYSM, EYPE). Therefore, it is unlikely that the roles of Lys and Arg in the membrane-damaging activity of Man-OVA are restricted to phospholipid binding. Several studies revealed that the incorporation of positively charged residues into membrane-damaging peptides could increase their pore-generation activity and the radius of the resulting pore [29,30,31]. Therefore, the functional contribution of Lys and Arg residues to the pore-forming ability of Man-OVA should be considered. In summary, our data reveal that the mannosylation of carboxyl groups generates a glycated OVA with membrane-perturbing activity, suggesting a novel method for the generation of functional OVA acting at the lipid-water interface at a physiological pH. A number of studies have paid attention to the membrane-interacting peptides and proteins, due to their applications as antimicrobial agents and mediators of drug release [32]. Accordingly, the biomedical applications of glycated OVA merit further investigation.

## 4. Materials and Methods

OVA (Grade VII, catalog number A7641, purity > 99%), calcein, 1-ethyl-3-(dimethylaminopropyl)-carbodiimide hydrochloride (EDC), *p*-aminophenyl α-dextro (d)-mannopyranoside, cholesterol (Chol), *N*-bromosuccinimide, TNBS, and phenylglyoxal, were purchased from Sigma-Aldrich Inc. (St. Louis, MO, USA), and 10,12-tricosadiynoic acid was obtained from Fluka (Buchs, Switzerland). EYPC, EYSM, PBPS, and EYPE were purchased from Avanti Lipids Polar Inc., and FPE was the product of Molecular Probes (Eugene, OR, USA). Sepharose 6B and PD-10 column were obtained from General Electric (GH) Healthcare Bio-Sciences Corp. (Piscataway, NJ, USA). Unless otherwise specified, all other reagents were of an analytical grade.

### 4.1. Preparation of Man-OVA

Man-OVA was prepared by coupling *p*-aminophenyl α-d-mannopyranoside with OVA through water-soluble EDC. OVA (6.8 mg) was added to 1.5-mL water (pH 4.75) containing a 50-, 150-, 300-, and 600-fold molar excess of *p*-aminophenyl α-d-mannopyranoside, and then a 100-, 300-, 600-, and 1200-fold molar excess of EDC, which was added to OVA dropwise, over a period of 30 min at room temperature. The reaction was allowed to proceed for 6 h, and the mixture was desalted by passage through a PD-10 column equilibrated with 0.01 M ammonium bicarbonate (pH 7.8). The protein fraction was pooled and lyophilized. The molecular weight of Man-OVA was determined using matrix-assisted laser desorption ionization-time of flight (MALDI-TOF) mass analyses. The absorption spectra of OVA (3.8 μM) and Man-OVA (3.8 μM) were measured using a Beckman Coulter DU640 UV/VIS spectrophotometer (Beckman Coulter Inc., Pasadena, CA, USA), and scanned from 400 to 200 nm.

### 4.2. Circular Dichroism (CD) Measurement

The CD spectra were obtained on a Jasco J-810 spectropolarimeter (JASCO corporation, Tokyo, Japan), with a cell path-length of 0.5 mm. The CD spectra were measured from 260 to 190 nm, and were obtained by averaging the signals of five scans.

### 4.3. Trp Fluorescence Measurement

The Trp fluorescence spectra of proteins were recorded with a Hitachi F-4500 Fluorescence spectrophotometer (Hitachi High-Technologies Corporation, Tokyo, Japan). A solution of OVA or Man-OVA in 10 mM Tris–HCl-0.1 M NaCl (pH 7.5) was measured with an excitation wavelength at 295 nm. The effect of temperature on the Trp fluorescence was monitored at the maximum emission at 334 nm. The data were presented as F/F_o_, F_o_, and F intensities at 30 °C, and at indicated temperatures from 30 to 70 °C. All values are the means of triplicate determinations.

### 4.4. Release of Entrapped Fluorescent Marker from Liposomes

The membrane-damaging activity of Man-OVA was determined by measuring the release of the liposome-entrapped, self-quenching fluorescent dye calcein. EYPC/EYSM (53/47, mol/mol), EYPC/EYSM/Chol (37/33/30, mol/mol/mol), PBPS/EYPE (50/50, mol/mol), or PBPS/EYPE/Chol (35/35/30, mol/mol/mol), were dissolved in chloroform/methanol (*v*/*v*, 2:1), and then the solvents were removed by evaporation. The lipid film suspended in 10 mM Tris–HCl-0.1 M NaCl (pH 7.5) was hydrated at a temperature above the phase-transition temperature, with vortexing. The multilamellar vesicles obtained in this way were frozen and thawed five times, and the mixtures were extruded 10 times through a 0.1 μM polycarbonate filter. Unencapsulated calcein was removed by gel filtration on a Sepharose 6B column (2 cm × 15 cm). The release of calcein was induced by adding aliquots of Man-OVA to a vesicle solution at 30 °C. Changes in fluorescence intensity were monitored with an excitation and emission wavelength at 490 and 520 nm, respectively. The total calcein release was achieved by the addition of 0.2% Triton X-100, and the data was presented as the percentage of total calcein release.

### 4.5. Lipid-Binding Experiments

FPE was incorporated at 2 mol % into indicated lipid compositions, which were prepared by extrusion through 100-nm pore size polycarbonate filters. Excitation was kept at 490 nm, and an emission intensity at 515 nm was monitored. The binding of proteins with FPE-containing vesicles causes a reduction or an increase in the membrane’s negative electrostatic potential, resulting in increased or decreased FPE fluorescence, respectively [21,22]. The data was corrected for dilution and the scatter contribution owing to the added protein solution and lipid vesicles. The data were fitted using a hyperbolic binding model: ΔF = ΔF_max_ [Protein]/(K_d_ + [Protein]), where ΔF and ΔF_max_ represent the fluorescence variation and maximum fluorescence variation, respectively. [Protein] is the protein concentration and K_d_ is the dissociation constant.

### 4.6. Colorimetric Phospholipid/Polydiacetylene Membrane Assay

The penetration of Man-OVA or OVA into phospholipid/polydiacetylene (PDA) vesicles was detected according to the methods described by Kolusheva et al. [25]. EYPC/EYSM/10,12-tricosadiynoic acid (21:19:60 molar ratio), EYPC/EYSM/Chol/10,12-tricosadiynoic acid (14.7:13.3:12:60 molar ratio), PBPS/EYPE/10,12-tricosadiynoic acid (20:20:60 molar ratio), or PBPS/EYPE/Chol/10,12-tricosadiynoic acid (14:14:12:60 molar ratio), was dissolved in chloroform/ethanol (1:1, *v*/*v*) and dried together in vacuo, followed by the addition of de-ionized water and sonication at 70 °C. The vesicle solution was then cooled to room temperature and kept at 4 °C overnight. The vesicles were polymerized using irradiation at 254 nm for 30–40 s, with the resulting phospholipid/PDA solution exhibiting an intense blue appearance. Vesicle sample for experiments were prepared at a concentration of 0.5 mM (total lipid) in 50 mM Tris–HCl, pH 7.5. The addition of Man-OVA or OVA, and the changes in the absorbance at 500 and 640 nm of vesicle solution, were measured.

A quantitative value for the extent of the blue-to-red color transitions within the vesicle solutions is given by the colorimetric response (%CR), which is defined as follows: %CR = [(PB_o_ − PB_1_)/PB_0_] × 100, where PB = *A*_640_/(*A*_640_ + *A*_500_) *A*_640_ and *A*_500_ are the absorbance measured at 640 nm and 500 nm, respectively. PB_o_ is the *A*_640_/*A*_500_ ratio of the control sample (before addition of protein), and PB_1_ is the value obtained for the vesicle solution after the addition of protein. All reported %CR values were averages of six independent measurements.

### 4.7. Acrylamide and Iodide Quenching

Quenching experiments were performed at an excitation wavelength of 295 nm, to ensure selective excitation of the Trp residues. The quenching of native and modified OVA (0.1 μM) fluorescence was conducted in the absence or presence of lipid vesicles (10 μM) in 10 mM Tris–HCl-0.1 M NaCl (pH 7.5). The fluorescence intensity was monitored at the emission maximum at 334 nm. For the quenching assays, small aliquots of 5 M acrylamide or KI were added to the protein solution in the absence or presence of liposomes. The quenching of fluorescence was corrected for dilution and the scatter contribution was obtained from the titration of liposomes with acrylamide or KI. The data were analyzed according to the Stern-Volmer equation: F_o_/F = 1 + K_sv_ [Q] [18], where F_o_ and F represent the fluorescence in the absence and presence of quencher, respectively. K_sv_ is the Stern-Volmer quenching constant, and a plot of F_o_/F versus [Q] gives a line with a slope corresponding to K_sv_.

### 4.8. Chemical Modification of Man-OVA

The modification of amino groups with TNBS was performed according to the procedure described in Chang et al. [33], with slight modifications. Man-OVA (2 mg) in 600 μL of 10 mM 4-(2-hydroxyethyl)-1-piperazineethanesulfonic acid (HEPES) (pH 7.5) was modified with a 20-fold molar excess of TNBS. The reaction was allowed to proceed for 60 min, and then desalted through a PD-10 column equilibrated with 0.01 M ammonium bicarbonate (pH 7.8). The incorporation of the trinitrophenyl group into Man-OVA caused an increase in the absorbance at 345 nm, and the extent of trnitrophenylation was determined spectrophotometrically, based on the molar absorption coefficient of 11,500 M^−1^·cm^−1^ at 345 nm [33].

The modification of Arg residues with phenylglyoxal was performed according to the procedure described in Chang et al. [34]. Man-OVA (2 mg) in 600 μL of 0.1 M sodium bicarbonate (pH 7.5) was modified with a 40-fold molar excess of phenylglyoxal. The reaction was allowed to proceed for 90 min, and then desalted through a PD-10 column equilibrated with 0.01 M ammonium bicarbonate (pH 7.8).

Trp residues of Man-OVA were modified with *N*-bromosuccinimide, according to the procedure described in Lundblad [35]. Man-OVA (2 mg) in 600 μL of 0.1 M sodium acetate (pH 4.0) was modified with a 10-fold molar excess of *N*-bromosuccinimide. The reaction was allowed to proceed for 60 min, and then desalted through a PD-10 column equilibrated with 0.01 M ammonium bicarbonate (pH 7.8).

### 4.9. Other Tests

Amino acid analysis, SDS-PAGE analysis, and HPLC analysis were performed in essentially the same manner as previously described [5,34]. All data are presented as the mean ± SD. Significant differences among the groups were determined using an unpaired Student’s *t*-test. A value of *p* < 0.05 was taken as an indication of statistical significance.

## 5. Conclusions

The results of the present suggest that glycation of carboxyl groups causes changes in the structural properties and membrane-interacting features of OVA, generating OVA with membrane-perturbing activities. Moreover, our data reveal a novel method for the preparation of functional OVA acting at the lipid-water interface at a physiological pH.

## Figures and Tables

**Table 1:** Acrylamide quenching constants determined from the Stern-Volmer plot.

Lipid Vesicles ^1^	OVA K_sv_ (μM)	Man-OVA K_sv_ (μM)
None	3.35 ± 0.04	5.34 ± 0.05
EYPC/EYSM	0.94 ± 0.02	0.61 ± 0.02
EYPC/EYSM/Chol	1.13 ± 0.03	0.72 ± 0.02
PBPS/EYPE	1.04 ± 0.03	1.34 ± 0.04
PBPS/EYPE/Chol	1.34 ± 0.04	0.08 ± 0.03

^1^ The used protein concentrations were 0.1 μM. Experiments were performed at a lipid-to-protein molar ratio of 100:1.

## Supplementary Materials

Supplementary materials can be found at www.mdpi.com/1422-0067/18/3/520/s1.

## Abbreviations

BSA	Bovine serum albumin
Chol	Cholesterol
CD	Circular dichroism
%CR	Colorimetric response
EDC	1-Ethyl-3-(dimethylaminopropyl)-carbodiimide hydrochloride
EYPC	Egg yolk phosphatidylcholine
EYSM	Egg yolk sphingomyelin
EYPE	Egg yolk phosphatidylethanolamine
FPE	*N*-(Fluorescein-5-thiocarbamoyl)-1,2-dihexadecanoylphosphatidylethanolamine
Man-BSA	Mannosylated BSA
Man-OVA	Mannosylated OVA
OVA	Ovalbumin
PBPS	Porcine brain phosphatidylserine
PC	Phosphatidylcholine
PDA	Polydiacetylene
PE	Phosphatidylethanolamine
PS	Phosphatidylserine
SM	Sphingomyelin
TNBS	Trinitrobenzene sulfonate
