# Supplementary material for: Ovalbumin with Glycated Carboxyl Groups Shows Membrane-Damaging Activity
Source: Int J Mol Sci. 2017 Feb 28;18(3):520. doi: 10.3390/ijms18030520 (PMC5372536; doi:10.3390/ijms18030520)
Supplement: Supplementary file 1 [file ijms-18-00520-s001.pdf]

Supplementary Materials: Ovalbumin with Glycated Carboxyl Groups Shows Membrane-Damaging Activity

Ching-Chia Tang, Yi-Jun Shi, Ying-Jung Chen and Long-Sen Chang

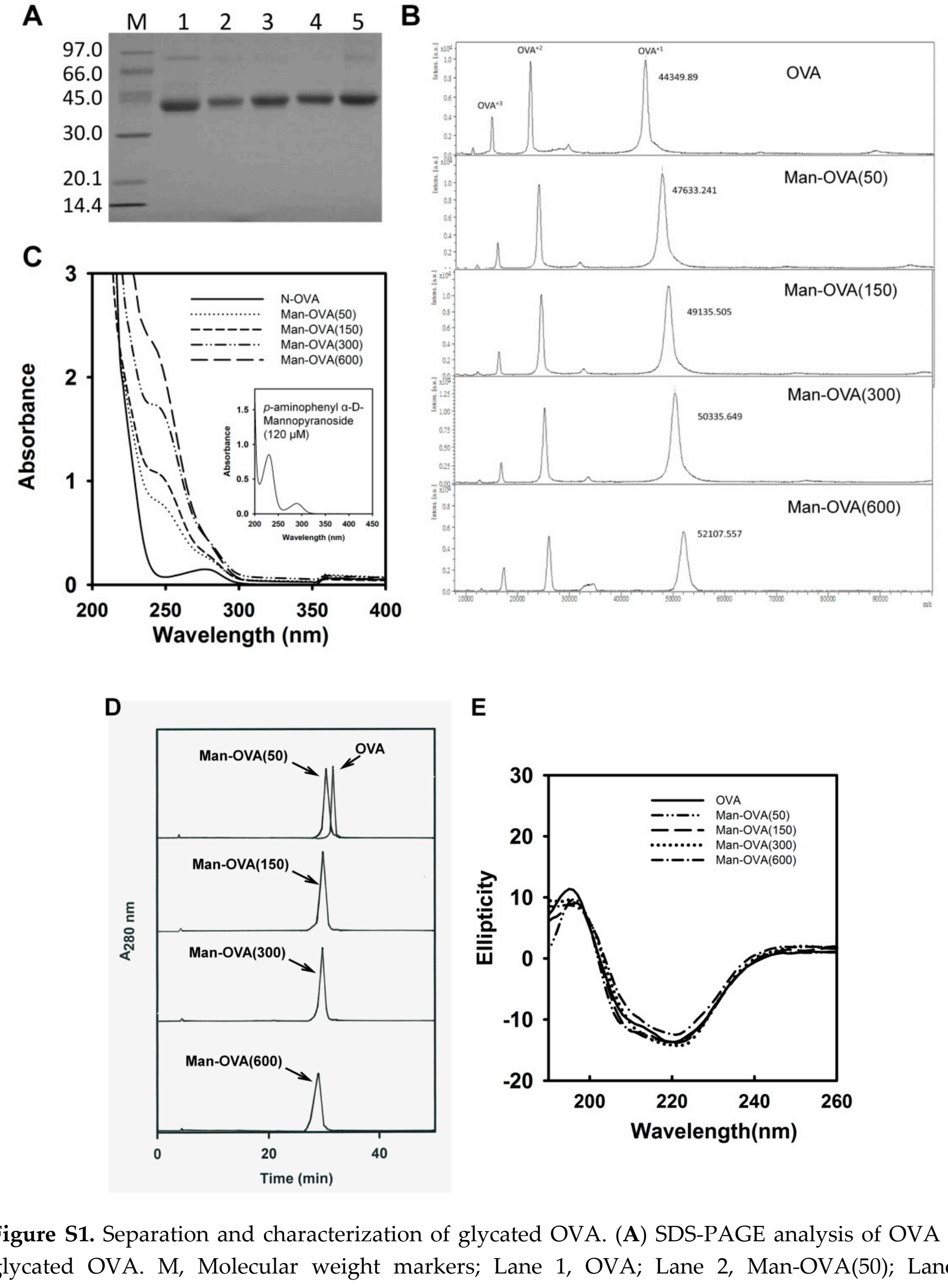

Figure S1. Separation and characterization of glycated OVA. (**A**) SDS-PAGE analysis of OVA and glycated OVA. M, Molecular weight markers; Lane 1, OVA; Lane 2, Man-OVA(50); Lane 3, Man-OVA(150); Lane 4, Man-OVA(300); Lane 5, Man-OVA(600); (**B**) Mass analyses of OVA and Man-OVA; (**C**) UV/VIS spectra of Man-OVA and OVA. (Inset) UV/VIS spectrum of 120 μM *p*-aminophenyl *α*-D-mannopyranoside; (**D**) OVA and Man-OVA were applied on a SynChropak RP-4 column (4.6 mm × 25 cm) equilibrated with 0.1% TFA and eluted with a linear gradient of 20%–50% acetonitrile for 50 min. Flow rate was 0.8 mL/min and the effluent was monitored at 280 nm. (**D**) CD spectra of Man-OVA and OVA.

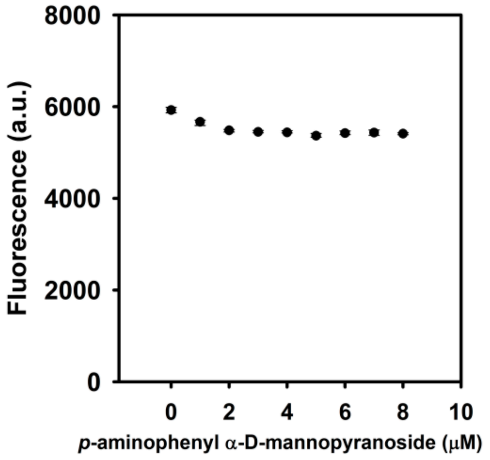

Figure S2. Effect of *p*-aminophenyl *α*-D-mannopyranoside on Trp fluorescence of OVA. OVA (0.1 μM) was titrated with varying concentration of *p*-aminophenyl *α*-D-mannopyranoside. The experiments were performed in 10 mM Tris-HCl-0.1 M NaCl (pH 7.5). Trp fluorescence intensity was measured using excitation wavelength and emission wavelength at 295 and 334 nm, respectively.
